# Identifying Medication Therapy Problems Related to Cognition Among Older Adults Followed by a Home-Based Care Team

**DOI:** 10.1093/geroni/igaa057.837

**Published:** 2020-12-16

**Authors:** Allison Levine, Erin Emonds, Marie Smith, Nathaniel Rickles, Richard Fortinsky, Alis Ohlheiser

**Affiliations:** 1 UConn Health, Farmington, Connecticut, United States; 2 VA Boston Healthcare System, Boston, Massachusetts, United States; 3 University of Connecticut School of Pharmacy, Mansfield, Connecticut, United States; 4 University of Connecticut School of Pharmacy, Storrs, Connecticut, United States; 5 University of Connecticut, Farmington, Connecticut, United States

## Abstract

Complications from dementia, depression, delirium (3Ds) and polypharmacy may accelerate patient decline. Cognitive vulnerabilities may be under-recognized and medication therapy problems (MTPs) overlooked, hindering optimal care. Clinical pharmacists on a multidisciplinary home-based care team (HBCT) being tested in a clinical trial were essential in identifying MTPs related to cognition. Medicare Advantage members >65 years old, living at home in Connecticut, with ICD-10 codes related to 3Ds were eligible. APRNs conducted in-home medication reconciliation along with medical and cognitive assessments. HBCT pharmacists assessed medication lists for MTPs related to indication, effectiveness, and safety (adverse events, interactions). After review by the HBCT APRN, geriatrician, and psychiatrist, salient pharmacist recommendations were forwarded to PCPs for consideration. Using retrospective analysis, MTPs and recommendations were classified based upon the Pharmacy Quality Alliance framework. MTP analysis included 105 patients enrolled from 2017-2019. We found 166 MTPs related to cognition, with a mean (SD) of 1.58 (1.35) (range 0-6) MTPs per patient. MTPs related to indication accounted for 34% (57/166) of total MTPs, of which 79% (45/57) were underuse and 21% (12/57) overuse; effectiveness represented 13% (22/166) of MTPs; safety represented over half (52%; 87/166) of total MTPs with benzodiazepines and anticholinergics commonly implicated. Common HBCT pharmacists’ recommendations included discontinuation (23%; 38/166) and dose reduction (19%; 32/166). MTPs related to cognition were found among the overwhelming majority (79%) of patients. This work is significant because it supports the value of pharmacists on multidisciplinary teams to address cognitively harmful medications, dementia treatment side effects, and untreated cognitive conditions.

